# Sensory integration therapy versus usual care for sensory processing difficulties in autism spectrum disorder in children: study protocol for a pragmatic randomised controlled trial

**DOI:** 10.1186/s13063-019-3205-y

**Published:** 2019-02-11

**Authors:** Elizabeth Randell, Rachel McNamara, Sue Delport, Monica Busse, Richard P. Hastings, David Gillespie, Rhys Williams-Thomas, Lucy Brookes-Howell, Renee Romeo, Janet Boadu, Alka S. Ahuja, Anne Marie McKigney, Martin Knapp, Kathryn Smith, Jacqui Thornton, Gemma Warren

**Affiliations:** 1 Centre for Trials Research, Cardiff University, 4th floor Neuadd Meirionnydd, Heath Park, Cardiff, UK; 2 School of Healthcare Sciences, Cardiff University, Ty Dewi Sant, Heath Park, Cardiff, UK; 3 Centre for Educational Development, Appraisal, and Research (CEDAR) University of Warwick, Coventry, UK; 4 Institute of Psychiatry Psychology and Neuroscience, King’s College London, London, UK; 5 Aneurin Bevan University Health Board, Newport, UK; 6 Department of Social Policy, London School of Economics, London, UK; 7 Mind Body Brain Connections Ltd, Threemilestone Industrial Estate, Truro, UK; 8 Centre for Developmental Psychiatry and Psychology, Monash University, Melbourne, Australia

**Keywords:** Sensory integration therapy, Autism spectrum disorder, Occupational therapy, Ayres, Sensory processing, School

## Abstract

**Background:**

Autism spectrum disorder (ASD) is a common lifelong condition affecting 1 in 100 people. ASD affects how a person relates to others and the world around them. Difficulty responding to sensory information (noise, touch, movement, taste, sight) is common, and might include feeling overwhelmed or distressed by loud or constant low-level noise (e.g. in the classroom). Affected children may also show little or no response to these sensory cues. These ‘sensory processing difficulties’ are associated with behaviour and socialisation problems, and affect education, relationships, and participation in daily life. Sensory integration therapy (SIT) is a face-to-face therapy or treatment provided by trained occupational therapists who use play-based sensory-motor activities and the just-right challenge to influence the way the child responds to sensation, reducing distress, and improving motor skills, adaptive responses, concentration, and interaction with others. With limited research into SIT, this protocol describes in detail how the intervention will be defined and evaluated.

**Methods:**

This is a two-arm pragmatic individually 1:1 randomised controlled trial with an internal pilot of SIT versus usual care for primary school aged children (aged 4 to 11 years) with ASD and sensory processing difficulties; 216 children will be recruited from multiple sources. Therapy will be delivered in clinics meeting full fidelity criteria for manualised SIT over 26 weeks (face-to-face sessions: two per week for 10 weeks, two per month for 2 months; telephone call: one per month for 2 months). Follow-up assessments will be completed at 6 and 12 months post-randomisation. Prior to recruitment, therapists will be invited to participate in focus groups/interviews to explore what is delivered as usual care in trial regions; carers will be invited to complete an online survey to map out their experience of services. Following recruitment, carers will be given diaries to record their contact with services. Following intervention, carer and therapist interviews will be completed.

**Discussion:**

Results of this trial will provide high-quality evidence on the clinical and cost effectiveness of SIT aimed at improving behavioural, functional, social, educational, and well-being outcomes for children and well-being outcomes for carers and families.

**Trial registration:**

ISRCTN14716440. Registered on 8 November 2016.

**Electronic supplementary material:**

The online version of this article (10.1186/s13063-019-3205-y) contains supplementary material, which is available to authorized users.

## Background

Difficulties in sensory processing (SP) are common in autism spectrum disorder (ASD) with prevalence estimates of 90–95% [1–3]. Such difficulties relate to hyper- or hypo-reactivity to sensory input and may occur due to impaired regulation of central nervous system arousal [4]. This hyper-reactivity may be associated with challenging behaviour such as aggression (e.g. to communicate discomfort with noise/touch), or additional “safe space” needs in the home [5]. Impaired sensory processing may also be associated with poor motor control impacting on participation in daily life. There is substantial potential burden related to sensory processing difficulties for children with ASD, their carers, and families, and also to the National Health Service (NHS) in terms of treating associated difficulties such as behaviour problems [6]. Sensory processing difficulties also pose significant challenges in mainstream education settings. It is plausible that interventions targeting sensory processing difficulties could result in improvements across behavioural, social, and educational dimensions.

A variety of potential play-based therapies have been proposed, with a clear distinction between sensory-based interventions (SBIs) and sensory integration therapy (SIT). SBIs are usually sensory strategies applied to the child or made available to the child for regulation of their reactivity within the home or school environment. Current research into the effectiveness of these SBIs is insufficient to recommend their use, especially if they are not individualised to the child [7].

SIT is a clinic-based approach that focuses on the therapist-child relationship and uses play-based sensory motor activities designed to improve sensation processing and integration [8]. SIT shows some promise as a potential therapy [9–11], but underpinning evidence is limited. In particular, some of the reported evaluations involved interventions either poorly defining criteria for SIT fidelity or indeed not meeting them at all [7]. Although SIT is currently offered by the NHS in some regions, the National Institute for Health and Care Excellence (NICE) reported that available evidence was of low quality and, therefore, insufficient to recommend treatment [12].

The key aims of this trial are to: 1) evaluate the effectiveness of manualised Ayres Sensory Integration® therapy (SIT) on behavioural problems and adaptive skills, socialisation, carer stress, quality of life, and cost; and 2) describe current usual care (UC) in trial regions and clearly differentiate this from the proposed intervention (SIT).

## Methods

### Primary objective

The primary objective is to determine the impact of SIT on irritability and agitation, as measured by the corresponding sub-scale of the Aberrant Behaviour Checklist (ABC) [13, 14].

### Secondary objectives

Secondary objectives are to evaluate:

i. The effectiveness of SIT for additional behaviour problems such as hyperactivity/non-compliance, lethargy/social withdrawal, stereotypic behaviour, and inappropriate speech
ii. The impact of SIT on adaptive skills, functioning, and socialisation
iii. Sensory processing scores post-intervention (i.e. at 6 months) as a potential mediator of any association observed between SIT and the primary outcome at 12 months
iv. Age, severity of SP difficulties, adaptive behaviour, socialisation, and comorbid conditions as potential moderators of any association between SIT and irritability/agitation, adaptive functioning (child), and carer stress
v. The impact of the intervention on carer stress and quality of life (QoL)
vi. Cost effectiveness of the intervention, including direct intervention costs, health, social care, education services, carer expenses, and lost productivity costs
vii. Fidelity, recruitment, acceptability, adherence, adverse effects, and contamination in a process evaluation conducted alongside the main trial. An internal pilot with specific progression criteria will assess the feasibility of proposed recruitment and trial retention rates and whether UC differs from expected provision of SIT.

### Study design

This is a two-arm individually randomised (1:1 ratio) effectiveness trial of SIT for children with ASD and SP difficulties in mainstream primary education (aged 4 to 11 years old). The comparator will be usual care (UC). Manualised SIT will be delivered in clinics meeting full fidelity criteria (structural equipment elements) [15]. The target is to recruit 216 children. Those allocated to the intervention group will continue to receive any care currently being received provided it does not contravene the eligibility criteria.

A process evaluation following Medical Research Council (MRC) guidance [16] will examine contamination, fidelity of intervention delivery, adherence, and any adverse effects. It will also include assessment of recruitment, retention, adherence, and intervention reach. Therapist and carer interviews will explore barriers/facilitators, adherence, therapeutic relationship, mechanisms of change, SP deficit, engagement in activities, and contamination.

### Eligibility criteria

Participants must:

i. have a diagnosis of ASD (as documented on medical and/or educational records), or have probable/likely ASD (defined as currently being assessed within the local ASD pathway);
ii. be aged 4–11 years at the start of the trial;
iii. plan to remain in mainstream primary education until the primary outcome time point (6 months post-randomisation, and end of intervention for SIT arm);
iv. have definite or probable SP difficulties defined as: (a) a definite dysfunction on at least one sensory dimension (all domains except social participation) and the total score on the Sensory Processing Measure (SPM) [17] or (b) at least a probable dysfunction on two or more sensory dimensions and the total score;
v. provide carer consent/child assent.

Other than the obverse of the inclusion criteria, participants will be excluded if they are:

i. currently undergoing or have previously undergone SIT;
ii. currently undergoing an intensive, comprehensive Applied Behaviour Analysis-based intervention.

### Recruitment process

Participants will be recruited from Child and Adolescent Mental Health Services (CAMHS)/paediatrics, occupational therapy, schools, and support/social services. The study will also be advertised on relevant websites (i.e. related charities’ websites) and via social media and a trial-specific website. It will also be possible for carers to make a self-referral.

### Informed consent

Potential participants will have a range of impairments and some may have a degree of intellectual disability. No child will be excluded on this basis, or due to other co-morbid conditions, provided all other inclusion criteria are met and exclusion criteria are not met. Informed consent from carers and assent from children will be sought. The child’s school may be asked for feedback on the child’s behaviour and will be asked to complete the Aberrant Behaviour Checklist irritability scale (ABC-I) at 6 months.

Assessment of fidelity and mentoring require sessions to be video recorded. Permission to video record the Autism Diagnostic Observation Schedule (ADOS) [18] will also be sought. Eligibility to participate in the trial is not, however, contingent on provision of consent to video record.

### Randomisation and blinding

Participants are assigned an identification number at consent. These are allocated sequentially for each site. Following screening, consent, and collection of baseline data, participants will be randomly allocated to usual care or SIT in a 1:1 ratio. Online randomisation will be carried out by the research team and will utilise minimisation with a random component used to allocate participants to the group that causes the least imbalance. Allocations will be minimised by site, severity of sensory processing difficulty, and sex of the child.

It will not be possible to blind recruiters to previous allocations. All data cleaning and manipulation prior to statistical analysis will be carried out blind to allocated treatment.

### Outcome measures

#### Primary outcome measures

**Fig. 1:**
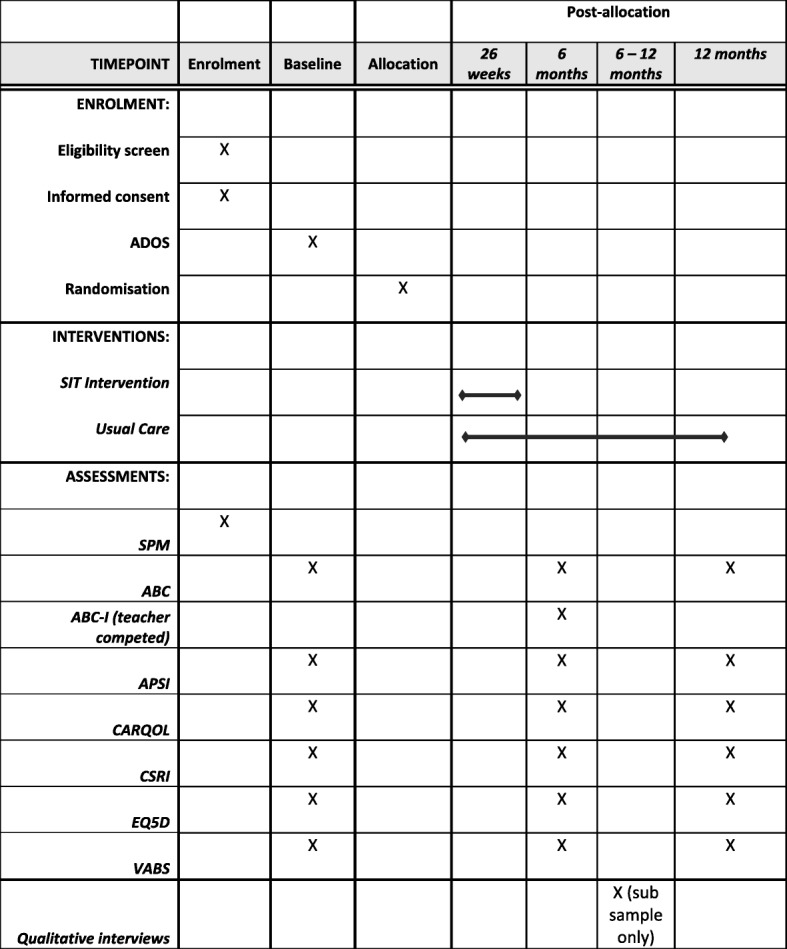
SPIRIT figure. Schedule of enrolment, interventions, and assessments. ABC Aberrant Behaviour Checklist, ABC-I Aberrant Behaviour Checklist irritability scale, ADOS Autism Diagnostic Observation Schedule, APSI Autism Parenting Stress Index, CARQOL carer quality of life, CSRI Client Service Receipt Inventory, EQ5D EuroQol 5D, SIT sensory integration therapy, SPM Sensory Processing Measure, VABS Vineland Adaptive Behaviour Scale

The primary outcome, to be measured at baseline and at 6 and 12 months post-randomisation (Fig. 1) is irritability/agitation as measured by the corresponding Aberrant Behaviour Checklist sub-scale (community version ABC-I: 15 items [13, 14]). This scale is completed by the parent/carer. The primary outcome time point is at 6 months post-randomisation (i.e. immediately post-intervention in the SIT arm). Teacher/teaching assistant ratings of ABC-I (assessed at 6 months post-randomisation only) will be measured to explore agreement between teacher and carer assessments and as way of measuring carer response bias.

#### Secondary outcome measures

All secondary outcomes will be measured at baseline and at 6 and 12 months post-randomisation (Fig. 1). These are all completed by the parent/carer.

Other problem behaviours will be measured using the remaining four ABC sub-scales [13, 19, 20].

Adaptive behaviours, socialisation, and functional skills change will be assessed using the Vineland Adaptive Behaviour Scale (VABS-II; parent/carer rating version) [21].

Carer stress will be assessed using the Autism Parenting Stress Index (APSI) [22].

Carer quality of life will be measured using two measures: the EQ5D 5 L [23] scale and CarerQol [24].

### Mediators

Scores on the SPM [17] will be assessed at screening and 6 months post-randomisation to determine whether any effects of the intervention on the primary outcome at 12 months (if observed) are mediated by the severity of SP difficulty post-intervention.

### Health economics measures

Detailed information on staff and non-staff inputs directly associated with the SIT intervention and UC will be recorded for each participant during the intervention period. Data on services and support external to the intervention will be collected at interview for each participant in the study at baseline (covering the previous 6 months) and at 6 and 12 months post-randomisation. The Client Service Receipt Inventory (CSRI) [25] will be adapted for use in this study to not only collect service and support data for the child but also data on health and social care services used by the child’s main carer. This will include carer out-of-pocket expenses and time taken off work to care for the child.

### Screening measure

**Fig. 2:**
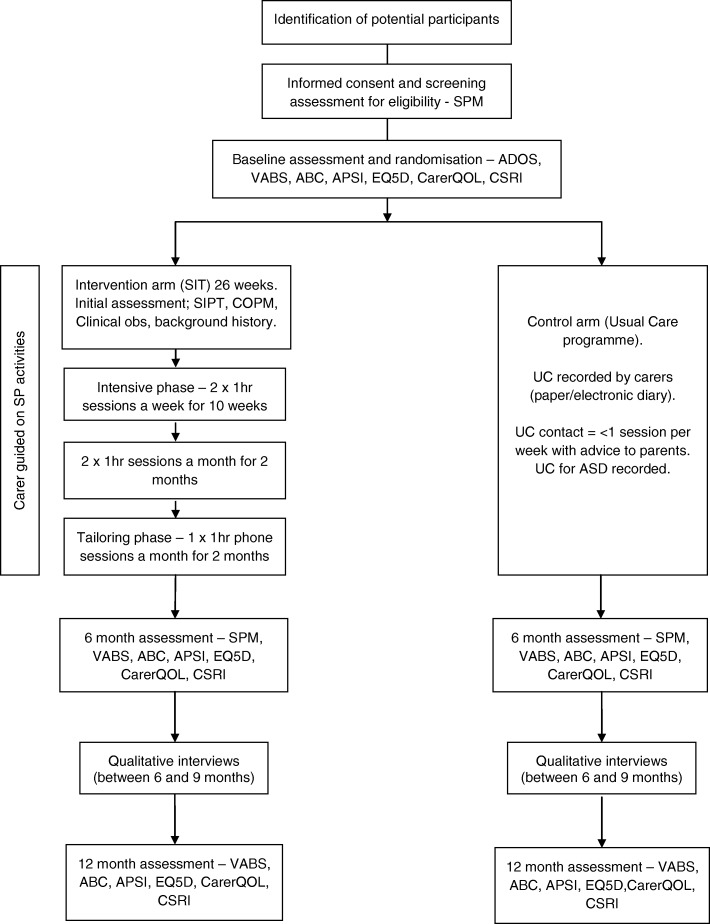
Summary flow chart. ABC Aberrant Behaviour Checklist, ADOS Autism Diagnostic Observation Schedule, APSI Autism Parenting Stress Index, ASD autism spectrum disorder, CarerQOL carer quality of life, COPM Canadian Occupational Performance Measure, CSRI Client Service Receipt Inventory, EQ5D EuroQol 5D, SIT sensory integration therapy, SIPT Sensory Integration and Praxis Test, SP sensory processing, SPM Sensory Processing Measure, UC usual care, VABS Vineland Adaptive Behaviour Scale

The SPM [17] is included at screening to confirm definite/probable sensory processing difficulties (Fig. 2). The measure provides norm-referenced standard scores for seven domains (visual, auditory, tactile, proprioceptive, vestibular, praxis, and social participation) and a total sensory systems score. Scores then fall into one of three interpretive ranges: typical, some problems, or definite dysfunction. For the purposes of the current trial, sensory processing difficulty is defined as either: 1) a definite dysfunction on at least one sensory dimension (defined as all domains except social participation) and the total score; or 2) at least a probable dysfunction on two or more sensory dimensions and the total score. Treating therapists will access these scores to aid with delivery of the intervention.

### Baseline measure

To characterise the recruited sample according to ASD symptoms, the ADOS will be included as a baseline measure. ADOS administrators will be trained to research standard and assessments will be video recorded. A sample of these recordings will then be used to ensure consistency in administration and coding between researchers.

### Data management

All assessments will be completed using web-based case report forms (CRFs). This is a secure encrypted system accessed by username and password. All data will be stored in accordance with the General Data Protection Regulation 2016. In the event that the web-based system is not accessible, paper CRFs will be used to record data. The data will then be inputted into the web-based system once it is accessible. A full data management plan detailing cleaning and quality control will accompany the protocol. We will make every effort to reduce loss to follow-up using strategies also outlined in the data management plan.

### Sensory integration therapy

Those allocated to the intervention arm will receive 26 1-h sessions of SIT [26, 27], delivered over 26 weeks: two sessions per week for 10 weeks (intensive phase), followed by two sessions per month for 2 months, and then one telephone session per month for 2 months (tailoring phase). A detailed assessment (SIT arm only) of sensory processing deficit will be undertaken (Sensory Integration and Praxis Test (SIPT) [28]) along with clinical observations post-randomisation. Following this assessment, the data will be scored to generate an SIPT report and a hypothesis developed as to the nature of the underlying sensory difficulty affecting function. In addition, background history, and the Canadian Occupational Performance Measure (COPM) [29] will be carried out. SIT uses the ‘just right’ challenge—a key principle of the sensory integrative approach—for each child and is therefore able to adjust the therapy to functional ability (as measured at baseline). Carers will be encouraged to observe or actively participate in sessions to facilitate engagement. Between sessions, carers may be given brief written guidelines of specific sensory-motor activities to support their child’s sensory integration. Success of these strategies will be discussed at the following session.

The intervention will be delivered by occupational therapists (typically NHS Band 7) trained in SIT and meeting fidelity criteria [15] in regional clinics which also meet fidelity. Initially, clinics will be located in South Wales and Cornwall with the potential for more to be included based on recruitment rates and therapist availability. For the duration of the trial, each intervention therapist will be paired with a mentor—a therapist independent to their clinic who is trained in both sensory integration and mentoring. The mentor will support the therapist in the assessment, interpretation, and intervention of the child. This is a critical part of the trial process that will help provide evidence of meeting intervention fidelity criteria. Mentoring sessions will be approximately 1 h long and provided ideally fortnightly during the first 2 months for the first participant during the intervention delivery phase, tapering to once per month or at least once every 6 weeks thereafter. A private, closed Facebook group for treating therapists to support each other will also be established. This is monitored by the research team to ensure that no personal information is included or discussed. Intervention therapists will provide therapy to participants recruited to the SIT arm only. Those participants receiving any form of usual care (such as provision of sensory strategies and/or face-to-face sessions delivered once per week or less) will be seen by occupational therapists not delivering SIT in the current trial.

After the participant has entered the trial, the therapist must remain free to give alternative treatment to that specified in the protocol, at any stage, if he/she feels it to be in the best interest of the participant.

### Fidelity assessment

Intervention delivery will be assessed using the Ayres Sensory Integration® Intervention Fidelity Measure [15]. The first two video recorded face-face sessions delivered to any participant for each therapist will be assessed purely to address any training issues at the earliest opportunity and to ensure ongoing fidelity can be rated. A sample of recorded sessions in the intensive phase will then be rated for fidelity by an independent SIT-trained therapist. Demonstration of adequate fidelity is defined as scoring 80/100 or above on the fidelity measure [15] across at least 80% of sessions sampled. An ‘effective’ dose for SIT has not yet been established; however, based on clinical experience and currently available evidence [7, 9–11] attending 13 of a possible 20 sessions delivered during the intensive intervention phase (two-thirds) is likely to indicate sufficient exposure.

Structural fidelity is assessed according to level of therapist training/qualifications, followed by a score of 85/110 for four areas: safety of the environment, detail and content of therapist-held records including therapist-carer collaboration in relation to goals set during therapy, physical space and equipment, and communication with carers.

We plan to identify suitable additional resources to use the video recording to look into fidelity of delivery in more detail. As part of this, we will include specific items to gauge the impact of non-specific therapist effects, using an adapted version of a tool developed for evaluation of psychosocial interventions for individuals with intellectual disability [30, 31].

### Comparator

Usual care (UC) will be recorded by carers in a diary format. The current standard care pathway is variable across the UK, ranging from minimal contact/no specific treatment targeted at sensory processing, to provision of manualised SIT in some regions. However, within the proposed trial sites, we estimate that usual care will be much less intensive than the 26-week programme detailed above, ranging from some provision of sensory strategies not meeting full fidelity criteria for SIT (and should not occur more frequently than once per week) to no specific treatment. Notes will be kept according to usual policy. Usual care for ASD will also be recorded more generally, including any contact with NHS services (e.g. speech therapy, paediatrics, and CAMHS).

Usual care for the current trial will be assessed and fully defined following a brief pre-recruitment survey of therapists, and discussions (e.g. as interviews or focus groups) with carers and occupational therapists. The potential for contamination, if participants recruited to the UC arm receive enhanced/additional support from clinicians who are aware of their participation in the trial, is acknowledged; thus, there will be an examination whether the UC received differs in any way from the provision mapped out as a result of the scoping focus groups.

### Internal pilot and progression criteria for full trial

An initial internal pilot phase will assess the feasibility of recruitment, retention to the intervention, and the nature of UC for sensory processing difficulties in the control arm.

Progression criteria are as follows:

1. Recruitment feasibility criteria will be met if at least 70% of those approached meet eligibility criteria for trial entry and at least 50% of those eligible are willing to be randomised. The proposed internal pilot end date is study month 18. Overall recruitment rates will be formally reviewed at this time point.
2. Once approximately 10% of participants have completed the post-intervention/6-month follow-up, carer-completed diaries will be qualitatively assessed to determine whether UC is sufficiently different from the SIT intervention for the full trial to continue. Broadly defined, this criterion will be met provided those in the UC arm do not receive any intervention meeting criteria for full SIT.
3. If drop-out at the 6-month post-randomisation time point exceeds 20%, the sample size calculation and associated implications for feasibility of recruitment will be re-assessed.
4. To confirm the accuracy of the sample size calculation and other features of the proposed design, an estimate of the following will be obtained: (a) proportion of participants providing primary outcome data; (b) standard deviation (SD) of the ABC-I at the primary outcome time point (post-intervention) in both SIT and UC groups; (c) intra-class correlation coefficient (ICC) of SIT therapists within participants for the ABC-I at the primary outcome time point (post-intervention, SIT arm only); (d) correlation between baseline and 6-month post-randomisation ABC-I. Sample size may be adjusted following these explorations.

### Collection of usual care data

A minimum of two scoping focus groups will be held prior to recruitment. Each will utilise a case analysis approach with clinicians providing treatment for sensory processing difficulties (one in each region). A small number of one-to-one telephone interviews may supplement focus group data as required. Focus groups will explore what is currently delivered/received as UC in the Health Boards/Trusts involved, and what if any difference exists in local provision and between regions (i.e. currently South Wales and England).

To develop a schedule for the focus groups, a brief survey will have been distributed to occupational therapist practice leads (OTs) in trial regions working with the trial population, via OT service leads.

Interviews with carers of children with ASD and sensory processing difficulties (parents from both South Wales and Cornwall) will be conducted. These will utilise a time-line facilitated process [31]. We will ask participants to focus on key points along a timeline, including ‘the beginning’, ‘diagnosis’, and ‘now’.

### Family carer and therapist interviews

Following the 6-month/post-intervention time point, interviews will be conducted with all SIT therapists (5–10 interviews) and a sample of family carers in both arms (anticipated to be 10–15 in each arm before data saturation).

Primary carers may choose to be interviewed alone or with other members of their family who are involved in day-to-day care. Participants will be asked to reflect on their experience of the intervention and the usual care activities that occurred alongside it, or their experience of usual care alone.

Therapists will be sampled to achieve variation in Health Board/Trust and regional centre and will be given the choice of telephone or face-to-face interviews. Family carers will be sampled to ensure maximum variation in terms of range of ASD and sensory symptoms, Health Board/Trust, and regional centre. Interviews will take place at a location of the interviewee’s choice, often their home, or over the phone.

The interview topic guides will be developed from a review of previous research, guides used by the research team in similar studies, and with input from the multi-disciplinary research team and family carer advisors to avoid bias in topic selection and wording of questions. The topic guide will be piloted and refined as necessary. Interviews will be recorded and transcribed.

### Safety reporting

No adverse events are expected. However, adverse events will be collected, recorded, and reported in accordance with Good Clinical Practice and the requirements of the research ethics committee. The Chief Investigator may carry out urgent safety measures if appropriate to protect participants from immediate harm.

### Sample size determination

We will recruit 216 participants in total (108 allocated to usual care, 108 allocated to the SIT intervention). This will provide 90% power at the 5% significance level (two-sided) to detect a standardised effect size of 0.5, allowing for 20% loss to follow-up at the primary outcome time point (6 months post-randomisation).

Our effect size is based on means and SDs of the ABC-I in relevant populations found in the literature [19, 32, 33]. This literature also suggests that a 25% relative difference represents a clinically meaningful difference on the ABC-I. Findings from the internal pilot will aid in confirming the accuracy of the assumptions behind the sample size calculation and could potentially lead to this being adjusted.

### Main analysis

The primary analysis will be based on the modified intention-to-treat (MITT) analysis population, and will estimate the between-group mean difference in the ABC-I at 6 months using linear regression, adjusting for baseline ABC-I, recruitment site, severity of SP difficulty, and sex of the child. If appropriate, therapist clustering will be accounted for using mixed models. Secondary outcomes will be analysed similarly.

### Sub-group analysis

Sub-group analyses will be conducted, exploring any differential intervention effects by site, region, age, sex of the child, severity of SP difficulties, adaptive behaviour, socialisation, motor skills, and comorbid conditions. This will be carried out by repeating the primary analysis but including each sub-group as an explanatory variable along with a sub-group × treatment arm interaction. Sub-group analyses will also be performed on carer stress.

### Impact of missing data and non-adherence

The impact that non-adherence to the intervention has on the intention-to-treat (ITT) findings will be investigated by estimating the complier-average causal effect (CACE) for the primary and secondary outcomes [34].

While the main trial analysis will be based on an MITT analysis population, sensitivity analyses will be carried out exploring the impact that missing data may have had on trial findings. Where outcome data are missing due to drop-out/loss to follow-up, these will be assumed to be missing at random given observed data (MAR), and multiple imputation will be used to achieve a full ITT analysis population. Additional sensitivity analyses will be conducted using joint modelling approaches (e.g. selection and/or pattern mixture models) to explore departures from an MAR assumption [35].

### Mediation analysis

Exploratory mediation analyses will be conducted to examine whether any effect of the intervention on behavioural problems at 1 year (all ABC subscales) is mediated through an effect on sensory sensitivities immediately post-intervention. The analyses will control for baseline measures of behavioural problems and SP difficulties to minimise any residual confounding between mediator and outcome [36]. Additional analyses will be conducted to explore the association between measures collected as part of the process evaluation and primary/key secondary outcomes. As the majority of process evaluation measures will only be collected for participants allocated to the SIT arm, the analysis will be purely associational and therefore hypothesis generating in nature.

### Exploratory analysis

Given the variability in the usual care that we are likely to see, we will conduct analyses using participants in the UC arm only that explore the association between different types of usual care and clinical outcomes. Parameters we will use to characterise different types of usual care will include number of treatment contacts, therapist experience/level of training, and type of difficulty for which the therapy is intended. Regression models will be fitted using our primary and secondary outcomes, and the therapy characteristics/parameters as explanatory variables. Variables that confound the relationship between therapy characteristics and outcome (e.g. age, severity of SP difficulty) will be investigated and controlled for in the models. The interpretation of the findings from these analyses will reflect the exploratory nature of this work and will be purely associational (that is, without ascribing cause).

A statistical analysis plan will provide further detail on analytical methods we will be using for the analysis of trial outcomes, and will be finalised prior to the end of recruitment.

### Qualitative analysis

Qualitative data will be analysed by the qualitative team using thematic analysis [37]. We will search across the data set to find repeated patterns of meaning, and identify key themes and sub-themes. We will identify contradictory data as points of contrast as well as similarities to understand uptake and engagement with the intervention. Vital measures will be put into place to ensure validity and reliability. Double coding will be carried out until consensus is reached. Data will be managed using qualitative coding software (such as NVivo10). This qualitative component has been designed using the principles of the Critical Appraisal Skills Programme qualitative checklist to ensure the quality of qualitative research [38].

### Health economic analysis

A within-trial health economic analysis will be conducted from a health and personal social services (NHS and PSS) perspective. The health economic analysis will be carried out on an ITT basis. The main analyses will compare cost and cost effectiveness at 6-month follow-up of SIT compared with UC. Total and mean costs for the SIT and UC group will be reported in a disaggregated format. Total and mean severity outcomes (ABC-I) will be reported for the intervention and control groups. The difference in mean scores between the two groups will be assessed with appropriate statistical tests. The difference in mean costs for the treatment groups will be analysed using regression analysis and bootstrapping. NHS and PSS costs (or societal costs in the secondary analyses) over the 6 months will be regressed on treatment allocation, baseline ABC-I, site, severity of SP difficulty, and baseline costs. We will account for clustering in the analysis.

An incremental cost-effectiveness ratio (ICER) will be calculated, defined as the difference in mean costs divided by difference in mean ABC-I as: the incremental cost per participant achieving a significant improvement in mean ABC-I score from an NHS/PSS perspective. Results will be plotted on a cost-effectiveness plane. Bootstrapping will be used to estimate a distribution around costs and behavioural outcomes and to estimate the confidence intervals around the ICER. Cost-effectiveness acceptability curves (CEAC), a recommended decision-making approach to dealing with uncertainty, will be generated by plotting these probabilities for a range of values of the ceiling ratio. Sensitivity analysis will be used to explore the sensitivity of the results from using a broader societal perspective (including NHS/PSS costs, education service costs, carer expenses, and lost productivity) than a narrower NHS/PSS perspective preferred by the NICE reference case [39]. Additional sensitivity analyses will build on results of the sub-group analyses.

### Trial management

A Trial Management Group (TMG) will meet 4–6 weekly and will include all investigators and the trial project team to discuss trial progression and key management issues. An Independent Data Monitoring Committee (IDMC) and Trial Steering Committee (TSC) will also be convened and will meet annually. The IDMC will comprise of a statistician as an independent chair and two other experts in the field. The IDMC will monitor data and make recommendations to the TSC on whether there are any ethical or safety reasons why the trial should not continue. The TSC will comprise of an independent Chair with expertise in trials of occupational therapy, an independent ASD expert, an independent statistician, and a health economist. The co-Chief Investigators, statistician and trial manager will be observers at each group. The TSC will provide overall supervision for the trial and provide advice through its independent chair and will advise the National Institute for Health Research whether the trial should continue following the results of the internal pilot. TSC and IDMC members will be required to sign up to the remit and conditions set out in a Charter.

### Quality control and assurance

A clinical trial risk assessment has been used to determine the intensity and focus of central and on-site monitoring activity. Low monitoring levels will be employed and fully documented in the trial monitoring plan.

Investigators should agree to allow trial-related monitoring, including audits and regulatory inspections, by providing direct access to source data/documents as required. Participant consent for this will be obtained. Findings generated from on-site and central monitoring will be shared with the Sponsor, Chief Investigator, Principal Investigator, and local R&D.

### Audits and inspections

The trial is participant to inspection by the Health Technology Assessment (HTA) programme as the funding organisation. The trial may also be participant to inspection and audit by Cardiff University under their remit as sponsor.

### Dissemination

All publications and presentations relating to the trial will be detailed in the publication policy which will be drafted and authorised by the TMG.

## Discussion

This trial will address the question: ‘What is the clinical and cost effectiveness of sensory integration therapy for children with autism spectrum disorder?’ As part of this unique trial design, we will also be including monitoring to ensure fidelity of intervention delivery as well as supervision/mentoring for therapist support. We believe this research will benefit the NHS in terms of providing clear evidence regarding the clinical effectiveness and cost effectiveness of this type of intervention, thereby informing clinical practice for this population. We also strongly believe that children and their families will benefit from receiving treatment informed by a more robust evidence base, whether or not SIT itself is effective. Furthermore, if SIT is effective, the proposed intervention could improve behavioural, functional, social, educational, and well-being outcomes for children and well-being outcomes for family carers. Sub-group analyses will also help to determine which children and families would be most likely to benefit, thereby maximising cost-effective roll-out.

## Trial status

The trial is sponsored by Cardiff University (Research and Innovation Services, RIScentraloperations@cardiff.ac.uk) and is currently on-going and open to follow-up. Recruitment commenced in July 2017 and is anticipated to end in Spring 2019. This manuscript has been drafted according to version 5.0 (11.04.2018) of the trial protocol. The protocol has been written according to the Standard Protocol Items: Recommendations for Interventional Trials (SPIRIT) statement (Additional file 1), the intervention according to the template for intervention description and replication (TIDieR) checklist, and the final report will follow the Consolidated Standards of Reporting Trials (CONSORT) statement.

## Additional file

Additional file 1: SPIRIT checklist. (DOC 122 kb)

## Abbreviations

ABC: Aberrant Behaviour Checklist
ABC-I: Aberrant Behaviour Checklist irritability scale
ADOS: Autism Diagnostic Observation Schedule
APSI: Autism Parenting Stress Index
ASD: Autism spectrum disorder
CACE: Complier-average causal effect
CAMHS: Child and Adolescent Mental Health Services
CarerQoL: Carer quality of life
CEAC: Cost-effectiveness acceptability curves
CONSORT: Consolidated Standards of Reporting Trials
COPM: Canadian Occupational Performance Measure
CRF: Case report form
CSRI: Client Service Receipt Inventory
EQ5D: EuroQol 5D
ICC: Intra-class correlation coefficient
ICER: Incremental cost-effectiveness ratio
ITT: Intention-to-treat
MAR: Missing at random
MITT: Modified intention-to-treat
MRC: Medical Research Council
NHS: National Health Service
NICE: National Institute for Health and Care Excellence
OT: Occupational therapy
PSS: Personal social services
QoL: Quality of life
SBI: Sensory-based intervention
SD: Standard deviation
SIPT: Sensory Integration and Praxis Test
SIT: Sensory integration therapy
SP: Sensory processing
SPIRIT: Standard Protocol Items: Recommendations for Interventional Trials
SPM: Sensory Processing Measure
TIDieR: Template for intervention description and replication
UC: Usual care
VABS: Vineland Adaptive Behaviour Scale
